# *Gnathostoma* infection after ingestion of raw fish is a probable cause of eosinophilic meningitis in the Brazilian Amazon

**DOI:** 10.1590/0037-8682-0434-2023

**Published:** 2024-03-04

**Authors:** Carlos Graeff-Teixeira, Dayse Souza de Pauli, Carlos Alexandre Martins Zicarelli, Vanessa Fey Pascoal, Estefany de Paula Paiva-Novaes, João Pedro Sarcinelli Chagas, Betina Bolina Kersanach, David Jamil Hadad, Letícia Karolini Walger-Schultz

**Affiliations:** 1 Universidade Federal do Espírito Santo, Núcleo de Doenças Infecciosas, Centro de Ciências da Saúde, Vitória, ES, Brasil.; 2 Hospital Evangélico, Londrina, PR, Brasil.; 3 Pontifícia Universidade Católica do Rio Grande do Sul, Escola de Ciências da Saúde e da Vida, Porto Alegre, RS, Brasil.; 4 Escola Superior São Francisco de Assis, Santa Teresa, ES, Brasil.

**Keywords:** Gnathostoma, Eosinophilic meningitis, Amazon

## Abstract

We report a case of eosinophilic meningitis associated with the ingestion of raw fish (*Cichla* sp.) from the Brazilian Amazon, likely caused by *Gnathostoma*. A 36-year-old male visited Juruena river on a fishing trip. After 50 days, the patient presented with an intense frontal headache. A cerebrospinal fluid examination revealed 63% eosinophilia. Another individual who ingested raw fish developed linear dermatitis on the abdominal wall. Anti-*Gnathostoma* serum antibodies were detected, and the patient made a full recovery after treatment with corticosteroids and albendazole. To date, autochthonous *Gnathostoma* spp. infections in Latin American countries have only caused linear panniculitis. This report raises awareness of gnathostomiasis-causing meningitis.

## INTRODUCTION

Eosinophilic meningitis (EoM) is usually caused by parasites^1^. In Thailand and other Asian countries, a nematode of the genus *Gnathostoma* is the second most frequent cause of EoM after *Angiostrongylus cantonensis* ^2^. When *Gnathostoma* infective third-stage larvae are ingested with undercooked intermediate or paratenic hosts, especially fish or other animals, the larvae migrate systemically. Lesions may subsequently develop in several organs and tissues, with the skin and central nervous system (CNS) being particularly affected^2^.

Similar to other countries in the Americas (Mexico, Colombia, Ecuador, and Peru), patients in Brazil have previously developed only cutaneous linear or nodular inflammatory lesions, as concluded after a careful review of the Cochrane Library, LILACS, SciELO, MEDLINE, PubMed, and PubMed Central databases. However, the present case report highlights the first autochthonous suspected case of EoM caused by gnathostomiasis in the Americas.

## CASE REPORT

**FIGURE 1:**
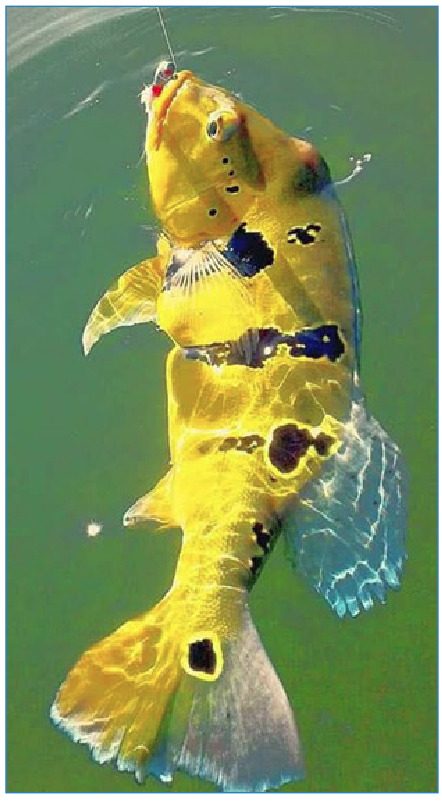
*Tucunaré* fish (*Cichla* spp.) was consumed raw in a “*sashimi”* preparation by the present patient and his group of friends and relatives during an ecotourism adventure in Juruena River (Tapajós River mesoregion) in Amazon region.

A 36-year-old Caucasian businessman and resident of Paraná State, southern Brazil, went on a fishing trip to the Juruena River in August 2017 with relatives and friends. The site visited was on the border between the Brazilian States of Amazonas (municipality of Apuí) and Mato Grosso (municipalities of Alta Floresta and Paranaíta), next to Jacareacanga and a locality of Barra de São Manoel (Pará State; latitude 7-8° South and longitude 58° West). Raw *Tucunaré* fish (*Cichla* spp.) were prepared in sashimi dishes containing wasabi and ginger on August 2^nd^ (Figure 1). Exposed individuals other than the present patient reported acute diarrheal episodes in early September. These episodes were treated with nitazoxanide (NTZ; 500 mg twice daily) for a period of three days. Despite the absence of diarrhea, the patient self-medicated with NTZ. On September 14^th^ (43 days post-exposure, PE), the patient presented with unusual fatigue, precordial palpitations, and dyspnea associated with habitual intense physical activity. The patient was an active triathlete. 

**FIGURE 2:**
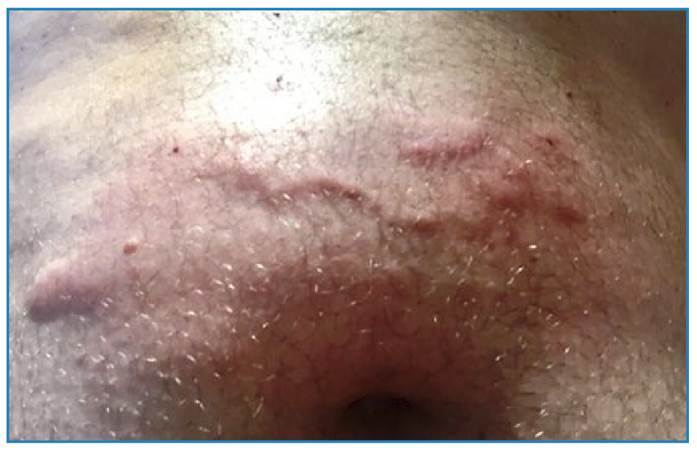
Nodular-linear migratory panniculitis at the supra-umbilical abdominal wall in a relative of the present patient who had the same exposure to *Gnathostoma* spp. larva after eating raw *Tucunaré* fish. A possible relapse of this cutaneous lesion was documented 5 years later and it subsided after retreatment with albendazole.

On September 21^st^ (50d PE, day 1), the patient reported the onset of intense and continuous frontal headache. The pain did not radiate from the original site and it was resistant to analgesic medication. Over the next 24 hours, the patient’s headache worsened, particularly with light exposure and head movements. There was no relief from the headache (day 2), and other possible clinical presentations, such as fever, chills, hyporexia, weight loss, vomiting, abdominal pain, or diarrhea were not noted. The following day, the patient visited the emergency department and reported a severe headache. No abnormalities were observed on physical examination, except for mild neck stiffness. Peripheral blood and cerebrospinal fluid (CSF) examinations were performed on September 22^nd^ and 23^rd^ (days 2 and 3) (Table 1). Magnetic resonance imaging of the head revealed no abnormalities. Considering the possibility of parasitic CNS infection, the patient was treated with albendazole (400 mg, 8/8 h) and dexamethasone (4 mg, 6/6 h). The patient’s headache subsequently improved, and he was discharged without any sequelae (day 10). The patient was prescribed albendazole for 21 days and oral corticoids for three months. Serum and CSF antibodies were not detected by IgG ELISA using *A.cantonensis* female worm crude antigens. The hypothesis of *Gnathostoma* infection was established after reviewing the exposure history and a report of linear dermatitis in the abdomen of another exposed individual (Figure 2). In May 2018, anti-*Gnathostoma* antibodies were detected in the CSF of the patient but not in his serum, according to an immunochromatographic test kindly provided by Khon Kaen University (Thailand).

**TABLE 1:** Results from blood and cerebrospinal fluid examinations from a patient with meningitis after ingestion of raw fish from the Amazon rainforest, Brazil, 2017.

Examinations		September 22	September 23
Blood	RBC^a^/mm^3^	5.2	4.7
	Hb^b^ (g/dL)	15.0	14.0
	HCT^c^ (%)	45.7	41.3
	WBC^d^/mm^3^	12,400	7,220
	Neutrophils	9,808 (79%)	3,877 (54%)
	Eosinophils	589 (4%)	1,213 (16%)
	Lymphocytes	1,538 (12%)	1,617 (22%)
Cerebrospinal Fluid	Color	Clear	
	Cells/mm^3^	496	
	RBC/mm^3^	43	
	Neutrophils	7%	
	Eosinophils	63%	
	Lymphocytes	30%	
	Lactic Acid	2.2	
	Protein	138	
	Glucose	53	
Serum	Glucose	92	

a Red blood cells; ^b^Hemoglobin; ^c^Hematocrit; ^d^White blood cells.

## ETHICS

The patient and relatives approved the study, provided written consent, and fully collaborated for the report, which was undertaken under the institutional (HUCAM) ethics committee permission CAAE 53085021.0.0000.5071.

## DISCUSSION

Since 2012, only five autochthonous human infections involving skin^3^ ^,^ ^5^ or eye^6^ lesions have been reported in Brazil. The present report on EoM caused by gnathostomiasis autochthonous to the Brazilian Amazon region serves to raise the awareness of physicians and health authorities regarding this health risk among the population. Specific preventive recommendations must also be made for the general population, particularly for those involved in ecotourism and recreational fishing. Furthermore, adequate examination of the CSF cellularity to detect eosinophils, access to diagnostic tools, and updated management guidelines are required for the best possible management of affected patients.

There are 20 recognized species of *Gnathostoma* ^7^ six of which have caused human infections worldwide^8^ ^,^ ^9^. *G.spinigerum* is the most common causative agent of cerebral gnathostomiasis in Asia. The identification of *G. spinigerum* in the American continent has been investigated and is currently under revision^7^. There is no evidence that *G.spinigerum* occurs in the New World. *G.binucleatum* is associated with human infections and skin lesions in Latin American countries, especially in Mexico^9^. In addition to *G. binucleatum*, other species have also been detected in natural animal hosts in South America. These include *G. turgidum* and *G. americanum* ^7^, neither of which is associated with human infections.

The occurrence of gnathostomiasis outside well-established endemic areas in Southeast Asia and Mexico is linked to the consumption of imported food^10^. However, both domestic and international ecotourism also represent a risk, with travelers returning from endemic areas able to carry an infection. The first reported Brazilian patient with gnathostomiasis returned from Peru^11^. All Brazilian patients, including the present patient, acquired infections during recreational fishing. In addition, the consumption of raw *Tucunaré* fish (*Cichla* spp.) is the most common source of infection in Brazil. The fishing sites were concentrated in two main areas within the neighboring hydrographic basins of the Amazon River (the Tapajós River mesoregion) and the Tocantins-Araguaia River in central Brazil. Since 2018, groups of amateur fishermen have shared both correct and incorrect information regarding the risk of gnathostomiasis associated with the consumption of raw *Tucunaré* on the Internet and on social media platforms. For example, 400 cases of gnathostomiasis in Brazil have been inaccurately reported as the cumulative number of gnathostomiasis cases in the country (https://pescaecia.com.br/2019/07/17/doenca-carne-tucunare/). In addition, many fishermen have strongly reacted to advice regarding gnathostomiasis posted at https://youtu.be/pp6rWteq1Gk and have denied any risk of the parasite.

In many Latin American countries along the Pacific coastline, fish meat is marinated in lime juice (*ceviche*), which is an ineffective method of killing nematode larvae^12^. Similarly, the spices used to prepare *sashimi* dishes were ineffective, as indicated by the present case and another case reported by Cornaglia and colleagues^3^.

In the present case, the detection of antibodies in the patient’s CSF and migratory and relapsing linear dermatitis in the abdomen of a co-exposed individual supported gnathostomiasis as an etiological diagnosis. It is noteworthy that NTZ was administered by the patient during the asymptomatic period prior to the onset of a severe headache. The patient reported by Vargas and colleagues^4^ also took albendazole shortly before the appearance of skin lesions. These two sets of observations support the ineffectiveness of anthelmintics in the prevention of gnathostomiasis. A well-known relapsing characteristic of cutaneous gnathostomiasis was observed in a co-exposed relative of the present patient. Relapse occurred five years after the first episode.

## CONCLUSION

Raised awareness of autochthonous gnathostomiasis as an additional cause of eosinophilic meningitis in South America will allow for extended studies on the transmission and morbidity of *Gnathostoma* species in the Amazon rainforest. This will also lead to the provision of adequate CSF examinations and molecular diagnostic tools, as well as epidemiological surveillance and notification procedures.
